# Trends in the Care of Diabetic Macular Edema: Analysis of a National Cohort

**DOI:** 10.1371/journal.pone.0149450

**Published:** 2016-02-24

**Authors:** Brian L. VanderBeek, Neepa Shah, Purak C. Parikh, Liyuan Ma

**Affiliations:** 1 Scheie Eye Institute, Department of Ophthalmology, University of Pennsylvania Perelman School of Medicine, Philadelphia, Pennsylvania, United States of America; 2 Center for Clinical Epidemiology and Biostatistics, Department of Biostatistics & Epidemiology, University of Pennsylvania Perelman School of Medicine, Philadelphia, Pennsylvania, United States of America; 3 Leonard Davis Institute, University of Pennsylvania Perelman School of Medicine, Philadelphia, Pennsylvania, United States of America; University of Florence, ITALY

## Abstract

**Purpose:**

To evaluate how the monitoring and treatment for diabetic macular edema (DME) has changed in a national sample.

**Design:**

Retrospective cohort study.

**Methods:**

Setting: Administrative medical claims data from a large, national U.S. insurer. Study population: Beneficiaries of a U.S. insurance company. Observation procedures: All incident cases of DME were found. Those in years 2002/3, 2006 and 2010 were followed for a 2-year observation period and those from 2009, 2010 and 2011 for a 1-year observation period. Main Outcome Measures: Types and frequencies of treatment were tallied and compared over each of the cohorts.

**Results:**

Two-year cohorts had 233, 251 and 756 patients in 2002/3, 2006 and 2010 respectively. One-year cohorts had 1002, 1119 and 1382 patients in 2009, 2010 and 2011, respectively. Both percentage of patients receiving therapy and number of treatments given increased across the 2-year cohorts for both focal laser and anti-vascular endothelial growth factor (anti-VEGF) (p<0.001). The highest use of anti-VEGF agents in any of the cohorts was in the 2011 1-year group that only averaged 3.78 injections. Focal laser was used 2.5x as frequently as anti-VEGF injections in the most recent cohorts with only a high of 14.0% of DME patients receiving anti-VEGF therapy in any of the cohorts.

**Conclusion:**

Regardless of treatment modality (laser or injection) DME patients received vastly fewer treatments than patients in randomized control trials. Despite the proven superior visual outcomes of anti-VEGF agents over focal laser in DME, focal laser was still used more frequently.

## Introduction

Macular edema is a significant cause of poor vision in those with diabetic retinopathy.[1] Until recently, focal laser had been the first line of therapy in treating diabetic macular edema (DME). Since biologics have emerged, however, multiple clinical trials have been performed and all have demonstrated that the addition of intravitreal anti-vascular endothelial growth factor (anti-VEGF) agents to the care of DME improves visual acuity far better than focal laser alone.[2–7]

Several studies have demonstrated the profound impact anti-VEGF agents have had on care, most notably in age-related macular degeneration (AMD). Curtis *et al*. used Medicare claims data to show that anti-VEGF use for AMD increased dramatically from 2006–2008, which paralleled an increase in office visits and a decrease in photodynamic therapy and thermal laser treatments.[8] Yet, concerns arose when later studies observed a high rate of anti-VEGF discontinuation, and also showed that even those who stayed on therapy received a lower frequency of injections than the monthly protocol thought to have optimal outcomes as demonstrated by major AMD randomized clinical trials.[9–11]

Similar worries were raised in DME when it was reported patients only received an average of 3.6 injections over the first year of care, far fewer than the 9–10 seen in most clinical trials.[12] However, by only examining the first year of treatment, it is unclear if the reported lower rate of injections resulted from a delay in treatment and that if care was observed on a longer time scale, an increased intensity of treatment would have been seen. Additionally, previous studies required the initiation of therapy for inclusion, excluding DME patients who were not treated due to having good vision or non-center involving edema.[12,13] The clinical trial-based evidence for treating both of these clinical variants of DME is less clear, potentially leading to an incomplete picture of resource utilization in the care of all DME patients.

Although treatment modality changes are the driving force in the evolution of DME care over the past decade, understanding how ancillary testing has also changed is important to inform future health policy and accurately predict required expenditures for disease care. One recent report has demonstrated a dramatic change in types of ancillary testing performed for various retinal diseases with an increase in optical coherence tomography (OCT) use, paralleling a decrease in fluorescein angiography (FA).[14] Although informative for broad policy planning, this study did not separate DME from other edema related diseases, nor did it longitudinally follow individual patients to determine the frequency of testing.

Multiple clinical trials have demonstrated that DME has high disease activity in the first year after diagnosis, but the need for treatment tapers greatly in the second year.[2–7] To date, no report has evaluated new DME patients in a non-clinical trial setting for up to 2 years. The aim of our study was to evaluate how the resource utilization for the diagnosis, monitoring and treatment of diabetic macular edema has changed within a national sample.

## Methods

### Dataset

Data was abstracted from the Clinformatics™ Data Mart Database (OptumInsight, Eden Prairie, MN), which contains the de-identified medical claims of all beneficiaries from a large managed care network in the United States. Included within the database are all outpatient medical claims (office visits, procedures, ancillary testing performed and medications given), as well as demographic data for each beneficiary during their enrollment in the insurance plan. The subset of data available for this study included all patients in the database from January 1, 2000 to December 31, 2012. Due to the de-identified nature of the database, the University of Pennsylvania’s Institutional Review Board deemed this study exempt from review.

### Subjects

Three cohorts were created to study the main outcome measures corresponding to the years 2002/3, 2006 and 2010. Incident cases of DME that occurred between January 1^st^ and December 31^st^ of each cohort year were included in the respective cohorts. Due to the overall lower volume of eligible participants in the early years of the database, the first cohort in the study, the 2002/3 cohort, allowed incident cases from both the full calendar years, 2002 and 2003. DME was defined as having an ICD9 code of 362.07, 362.53, 362.82 or 362.83 in conjunction with a code for ocular (362.01-.06) or systemic diabetes (250.xx) on the same office visit to an eye care provider. Use of ICD9 codes for the detection of diabetic macular edema has been validated previously.[15] The index date for each person was considered the first date DME was diagnosed. For inclusion into the study, individuals had to have at least 18 consecutive months in the plan prior to the index date and 24 consecutive months in the plan after the index date.

Individuals were excluded if at any time prior to the index date, they had a diagnosis of DME, any disease state that may be confused for DME or a disease that used diagnostic and treatment resources similar to DME including proliferative retinopathies, sickle cell disease, vein occlusions, pathologic myopia, retinoschisis, age-related macular degeneration, cystoid macular edema, all uveitides, and glaucoma. Since cystoid macular edema is a common complication of intraocular surgery, all patients with a code for an intraocular surgery within 90 days of index were also excluded. Lastly, to further ensure the use of resources being studied here were due strictly to the care of DME, any one who had a diagnosis code in the 24 months after the index date for a proliferative retinopathy, sickle cell disease, vein occlusion, retinoschisis, age-related macular degeneration, any uveitis or any glaucoma were also excluded. **Table 1** contains all diagnosis, procedure and drug codes used in the study.

**Table 1:** ICD-9, CPT and Drug codes used in this study.

**ICD-9 Codes**	**Disease**	**Codes**
	Diabetic Macular Edema	362.07, 362.53, 362.82, 362.83 + 250.xx, 362.01, 362.03, 362.04, 362.06
	Proliferative Retinopathies	362.02, 362.15, 362.16, 362.2x, 364.42, 365.63, 365.89
	Sickle Cell Disease	282.6
	Vein Occlusions	362.3x
	Separation of Retinal layers	362.4x
	Macular Degeneration	362.5x
	Uveitis	363.xx, 364.0x, 364.1x, 364.2x, 364.3x, 364.4x,
	Glaucoma	365.1x, 365.2x, 365.3x, 365.4x, 365.5x, 365.6x, 365.7x
**CPT codes**	**Procedure**	**Code**
	Office Visits	99201–99205, 99211–99215, 99241–99245, 92002–92014
	Fluorescein Angiography	92235
	Optical Coherence Tomography	92134, 92135
	Fundus Photography	92250
	Focal Laser	67210
	Subtenon Injection	68200
	Intravitreal Injection	67028
	Intraocular Surgery	650xx-653xx, 657xx-659xx, 66xxx, 670xx-672xx
**Drug Codes**	**Drug**	**Code**
	Bevacizumab	J3590, J9035, J3490
	Ranibizumab	J2778
	Steroids	J3300, J3301, J3302, and J3303

To make better comparisons to other studies and to best fully utilize the available data in the dataset, a second set of 1-year cohorts (2009–10, 2010–11 and 2011–12) was also created using identical inclusion and exclusion criteria as above. The lone difference from 2-year and 1-year cohorts was that the period of post-index date observation was decreased from 24 to 12 months. (For clarity purposes, all cohorts in the following text will be labeled to include the observation period to distinguish between 1- and 2-year cohorts.) Incident cases were again collected and followed in each of these cohort years to specifically analyze the most recent changes in treatment trends.

### Outcome measures

All patients were observed for the subsequent 24 months (or 12 months based on cohort) after index date regardless of when during the course of the cohort year an incident diagnosis of DME was made. For example, a beneficiary in the 2-year cohort diagnosed with DME on 12/27/2010 would be followed through 12/26/2012. Basic demographic information was collected on each patient at time of index date including age, race and gender. Rates of office visits, types and frequencies of treatment (focal laser, anti-VEGF injection, etc.) were the primary outcome measures. Patients that had a bilateral code in conjunction with one of the treatment codes were counted to have had 2 procedures. Secondary outcomes were types and frequencies of ancillary testing (FA, fundus photography, and OCT) used during the observation period. Patients were not allowed to have more than one of each of the separate ancillary tests on the same calendar day. Baseline and demographic characteristics were summarized using descriptive statistics (e.g. means and standard deviations for continuous variables such as age; percentages for categorical variables). Frequencies were compared using rate ratios. STATA^®^ 14 (College Station, Texas) software was used for all statistical analysis. Results of the analyses were considered statistically significant for p<0.05 (two-tailed).

## Results

**Fig 1:**
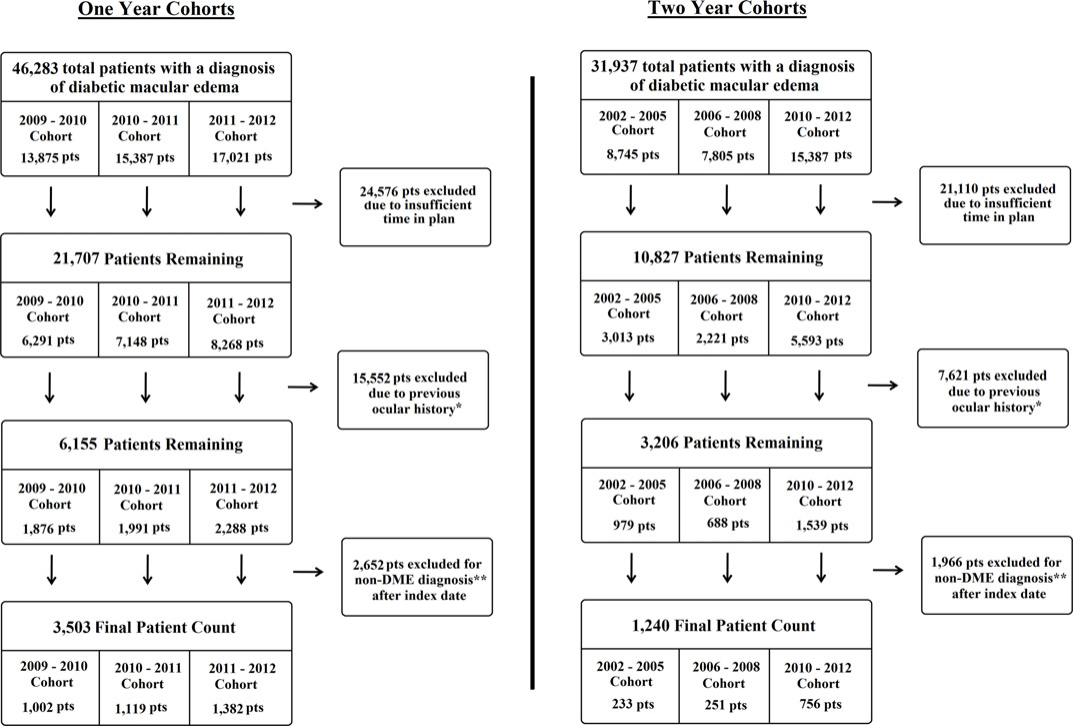
Flow chart for inclusion/exclusion criteria for each of the final cohorts. *Diagnoses included proliferative retinopathies, sickle cell disease, vein occlusions, pathologic myopia, retinoschisis, age-related macular degeneration, cystoid macular edema, all uveitides, glaucoma and intraocular surgery within 90 days of index date. **Diagnoses included proliferative retinopathies, sickle cell disease, vein occlusions, pathologic myopia, retinoschisis, age-related macular degeneration, cystoid macular edema, all uveitides, and glaucoma.

In total, 1240 patients met the strict inclusion/exclusion for the 2-year cohort study: 233, 251 and 756 in the 2002–5, 2006–8 and 2010–12 cohorts, respectively (see **Fig 1**). **Table 2** shows the baseline characteristics of the 2-year cohorts. The 2010–12 cohort was older than the 2002–5 cohort (p<0.001) and the percentage of “unknown” race was higher in 2002–5 compared to the other cohort years (p = 0.002). There were no other differences in race composition (p>0.05 for all comparisons) of the 2-year cohorts. The mean number of visits increased over the decade of observation with the 2010–12 cohort having the most visits at 3.77 (SD ±2.90) over the 2 years post index date (p = 0.054).

**Table 2:** Basic 2-year cohort demographics and office visit data.

	2002/3-2006	2006–08	2010–12	p-value
**Patients**	233	251	756	
**Age in years (SD)**	57.1 (12.7)	58.8 (12.1)	60.3 (12.6)	***<0*.*001***
**% Male (N)**	58.37% (136)	57.37% (144)	50.93% (385)	***<0*.*001***
**Race (N)**				***0*.*002***
Asian	3.86% (9)	5.18% (13)	3.31% (25)	
Black	16.74% (39)	17.93% (45)	21.43% (162)	
Hispanic	5.58% (13)	12.75% (32)	12.96% (98)	
White	62.66% (146)	60.16% (151)	56.75% (429)	
Unknown	11.16% (26)	3.98% (10)	5.56% (42)	
**Office Visits**				
Total Visits	787	838	2853	
Visit Range	1–14	1–16	1–21	
Avg Visits (SD)	3.38 (2.15)	3.34 (2.30)	3.77 (2.90)	0.054

Treatment frequency increased for both anti-VEGF and focal lasers in each cohort compared to baseline. (**Table 3**) Similarly the percentage of patients who received any treatment rose in each of the successive 2-year cohorts (p<0.001). Specifically, the percentage of patients who had focal laser therapy increased significantly in each successive cohort from 22.7% to 36.6% (p<0.001). Anti-VEGF use also increased from 0 to 14.6% of patients in the final cohort (p<0.001). Bevacizumab was the predominant anti-VEGF used during the study with ranibizumab (first used in 2010 for both sets of cohorts) accounting for less than 10% of injections and no aflibercept was given. Of the patients who did receive focal laser therapy, the average number of lasers received increased from 1.28 in the 2002–5 cohort to 1.99 in 2010–12 cohort (p<0.001). The 2006–8 cohort averaged 2.00 injections while the 2010–12 cohort averaged 3.90, but the small number of total injections (10) in the 2006–8 cohort prevented this difference from being statistically significant (2002–5 did not have any injections given). Of those receiving anti-VEGF injections, the 2010–12 cohort averaged 2.20 and 1.70 injections per year in the first and second years of the cohort respectively. Steroid injections, either subtenon or intravitreal, were rarely used throughout all the cohorts with only a high of 2.4% (n = 18) of the patients receiving a steroid treatment in the 2010–12 cohort.

**Table 3:** 2-year cohort data on treatment types and frequencies.

	2002/3-2005	2006–8	2010–12	p-value
**Patients (PT)**	233	251	756	
**Total Office Visits**	787	838	2853	
**Focal Laser Treatments**				
**% PT w/ focal laser (N)**	22.75% (53)	35.06% (88)	36.64% (277)	***<0*.*001***
**Total focal lasers performed**	68	158	552	
**Range of focals performed**	0–4	0–10	0–10	
**# Focals/focal PT (SD)**	1.28 (0.63)	1.80 (1.39)	1.99 (1.44)	***<0*.*001***
**# Focals/total visits (SD)**	8.64% (0.28)	18.85% (0.39)	19.34% (0.40)	***<0*.*001***
**Anti-VEGF Treatments**				
**% PT w/ Anti-VEGF (N)**	0% (0)	2.00% (5)	14.55% (110)	***<0*.*001***
**Total Anti-VEGF injections**	0	10	430	
**Range of injections performed**	0	0–3	0–15	
**# Injections/anti-VEGF PT (SD)**	0 (NA)	2.00 (1.00)	3.91 (3.21)	0.19
**# Injections/total visits (SD)**	0 (NA)	1.19% (0.11)	15.07% (0.36)	***<0*.*001***
**Steroid Injections**				
**% PT w/ steroid (N)**	0% (0)	1.20% (3)	2.38% (18)	***0*.*04***
**Total steroid injections**	0	3	31	
**Range of injections performed**	0	0–1	0–5	
**# Injections/steroid PT (SD)**	0 (NA)	1 (0.00)	1.72 (1.18)	0.31
**# Injections/total visits (SD)**	0 (NA)	0.36% (0.06)	1.09% (0.10)	***0*.*003***
**Any Treatment**				
**% PT with any treatment (N)**	22.75% (53)	35.86% (90)	40.48% (306)	***<0*.*001***

Due to the less restrictive inclusion criteria of requiring 12 months of follow up time (compared to 24 in the 2-year cohort), the number of incident DME cases was considerably higher for the 1-year cohorts (2009–10: 1002, 2010–11: 1119 and 2011–12: 1382) (**Fig 1**). The 1-year cohort treatment rates are seen in **Table 4**. When examining the specific treatment modalities, the percentage of patients receiving focal laser did not change over the 3 individual 1-year observation periods (p = 0.233), nor did the number of focal treatments received (p = 0.115). The percentage of patients who received an anti-VEGF agent did increase from 5.79% in the 2009–10 cohort to 14.04% in the 2011–12 cohort (p<0.001). Similarly the average number of injections increased across each cohort from 2.50 to 3.78 (p<0.001). Despite this increase in anti-VEGF use, no change in the percentage of patients who received any type of treatment was seen (2009–10: 34.13%, 2010–11: 37.80%, and 2011–12: 37.12%; p = 0.176), suggesting some focal laser use was replaced by anti-VEGF use in the more recent 1-year cohorts.

**Table 4:** 1-year cohort data on office visits, treatment types and frequencies.

	2009–10	2010–11	2011–12	p-value
**Patients (PT)**	1002	1119	1382	
**Total Office Visits**	2404	2778	3496	
**Avg # Office Visits (SD)**	2.40 (1.60)	2.48 (1.76)	2.53 (1.74)	***0*.*003***
	**Focal Lasers**			
**% PT w/ focal laser (N)**	31.84% (319)	33.87% (379)	30.68% (424)	0.233
**Total focal lasers performed**	539	637	671	
**# Focals/focal PT (SD)**	1.69 (0.97)	1.68 (0.88)	1.58 (0.88)	0.115
	**Anti-VEGF Injections**			
**% PT w/ Anti-VEGF (N)**	5.79% (58)	11.35% (127)	14.04% (194)	***<0*.*001***
**Total Anti-VEGF injections**	145	349	734	
**# Injections/anti-VEGF PT (SD)**	2.50 (2.09)	2.75 (2.08)	3.78 (3.19)	***<0*.*001***
	**Steroid Injections**			
**% PT w/ steroid (N)**	1.30% (13)	1.43% (16)	2.03% (28)	0.312
**Total steroid injections**	23	28	61	
**# Injections/steroid PT (SD)**	1.77 (0.93)	1.75 (1.18)	2.18 (2.16)	0.517
	**Any Treatment**			
**% PT with any treatment (N)**	34.13% (342)	37.80% (423)	37.12% (513)	0.176

Although less dramatic, ancillary testing also showed changes over the different 2-year observation periods (**Table 5**). OCT rates increased significantly in both the percent of patients who received the study (12.0% in 2002–5 to 70.6% in 2010–12 cohorts, p<0.001), and the frequency of testing that occurred (4.0% compared to 51.0% of total visits having an OCT in 2002–5 and 2010–12, respectively (p<0.001)). During the observation period, fluorescein angiography occurred in 39.1% of patients in 2002–5, 43.4% of patients in 2006–8, and 44.6% of patients in the 2010–12 cohort (p = 0.33). The FA rate per office visit also did not change with FAs done in 13.6%, 17.8% and 16.9% of office visits, respectively (p = 0.08). The mean number of FAs performed in patients who had at least one FA, however, did increase from 1.18 in 2002–5 to 1.43 in 2010–12 (p = 0.007). The percentage of patients who received fundus photography was stable over the 3 cohorts (47.6% in 2002–5, 45.8% in 2006–8 and 47.4% in 2010–12, p = 0.37), as was the fundus photography rate per office visit (20.6% in 2002–5, 21.6% in 2006–8 and 20.2% in 2010–12, p = 0.63).

**Table 5:** Data on ancillary study types and frequencies in the 2-year cohorts.

	2002/3-2006	2006–08	2010–12	p-value
**Patients (PT)**	233	251	756	
**Total Office Visits**	787	838	2853	
**Optical Coherence Tomography (OCT)**				
**% PT w/ OCT (N)**	12.02% (28)	48.61% (122)	70.63% (534)	***<0*.*001***
**Total OCTs performed**	33	224	1455	
**Range/PT**	0–3	0–13	0–19	
**OCTs/ OCT PT (SD)**	1.17 (0.48)	1.84 (1.63)	2.72 (2.63)	***<0*.*001***
**OCTs/total visits (SD)**	4.19% (0.20)	26.73% (0.44)	51.00% (0.50)	***<0*.*001***
**Fluorescein Angiography (FA)**				
**% Patients w/ FA (N)**	39.06% (91)	43.43% (109)	44.58% (337)	0.33
**Total FAs performed**	107	149	481	
**Range/PT**	0–4	0–5	0–6	
**FAs/ FA PT (SD)**	1.18 (0.55)	1.37 (0.73)	1.43 (0.83)	***0*.*007***
**FAs/total visits (SD)**	13.60% (0.34)	17.78% (0.38)	16.86% (0.37)	0.08
**Fundus Photography (FP)**				
**% PT w/ FP (N)**	47.64% (111)	45.82% (115)	47.35% (358)	0.37
**Total FPs performed**	162	181	576	
**Range/PT**	0–4	0–5	0–6	
**FPs/ FP PT (SD)**	1.46 (0.74)	1.57 (0.94)	1.61 (0.95)	0.13
**FPs/total visits (SD)**	20.58% (0.41)	21.60% (0.41)	20.19% (0.40)	0.63

## Discussion

The Diabetic Retinopathy Clinical Research Network’s Protocol I study was published in June of 2010 and was the first study to definitively show anti-VEGF injections (with or without supplemental laser) dramatically improved the visual acuity results for DME patients compared to laser alone.[16] The current study draws from a national sample to report how the care, treatment and health care resource utilization for DME has changed due to studies such as Protocol I and others that followed. As was expected, significant increases in the utilization of anti-VEGF treatments and OCT occurred over the 3 individual 2-year cohorts. Additionally, rates of office visits and fluorescein angiography all grew during the study with the highest levels of use in the 2010–12 cohort, but none of these trends reached significance.

Most surprising of the findings was that despite clinical trial results, focal laser was still the therapy of choice in both the most recent 2-year and 1-year cohorts with nearly 2–2.5 times as many patients receiving laser compared to anti-VEGF agents. Furthermore, contrary to what would be expected, the percentage of patients receiving focal laser therapy increased significantly across the 2-year cohorts (although this observation was not seen for the shorter 1-year cohorts). In conjunction with the increased laser usage was a similar increase in the frequency of use within those that received it. Despite the surge in focal laser use, however, the average was still far below the number of lasers received by participants at the 2-year mark in the laser arms of the DRCR network Protocol I study (3.0–3.7), suggesting a possible under treatment in these patients within this type of treatment.[16]

This study found that in its most recent 1-year cohort (2011–2012) of those treated with anti-VEGF agents, they received an average of 3.7 injections during the first year. This number corresponds well with the only other study to examine the frequency of anti-VEGF injections in a “real world” (i.e. outside of a clinical trial) setting for DME which found 3.6 injections in a similar group.[12] Although the average number of injections increased significantly from 2010–11 to 2011–12, the 3.7 injections seen in the 2011–12 would need a 2.5 fold spike in usage to match the average 9–10 seen in randomized clinical trials during the first year of treatment.[2,5,6]

One could postulate that despite the Protocol I results, the fact the FDA did not approve ranibizumab for DME treatment until the summer of 2012 may partially explain the overwhelming use of focal laser compared to anti-VEGF agents. One argument in favor of this idea was the low use (<10%) of ranibizumab injections across throughout the study. Further inquiries will be required to see how FDA approval impacts how frequently and which anti-VEGF agents are used for DME in more recent cohorts. In contrast to this theory however, it should also be noted that bevacizumab (consisting of >90% of injections in this study) has been shown by Lad and colleagues to be the initial therapy of choice in the U.S. at a rate of 2:1 over ranibizumab for AMD patients covering similar study years.[9] This demonstrates that treating physicians were comfortable with bevacizumab as a treatment and the lack of indication specific FDA approval was not a major hindrance to its use during the study period.

A second potential explanation for the lower injection frequency seen in the 1-year cohorts is a possible delay in therapy, where each patient would experience more injections in the second year of treatment if monitored over a longer period. To test this, a 2-year observation period was constructed in our study over which there was a reduction in the average number of injections from the first year to the second. This decrease contradicts the possibility of delayed therapy and further re-enforces the idea of possible under treatment in these patients.

With the intention of creating a comprehensive picture of the care given to all DME patients, this study chose to include all patients with new diagnoses of DME and not limit the study pool to only those who were treated. Interestingly, the rate of treatment increased dramatically from the 2002–5 cohort to the 2010–12 cohort. One possible explanation for this increase could be the increasing use of OCT’s on these patients. The ability to view subclinical edema in cross section may have spurred treatment in situations where previously the edema would have otherwise gone unnoticed or untreated (particularly as more practices switched from a time-domain OCT to a spectral-domain OCT showing much greater retinal physiologic detail).

Although the percentage of treated patients increased across the 2-year cohorts, somewhat surprisingly, no more than 41% of patients diagnosed with DME ever received treatment in any of the 1- or 2-year cohorts. Unfortunately, one limitation of all administrative medical claims studies is that chart level detail is not found within the claims data. Without specific exam and ancillary test findings, it is impossible to determine what percentage of patients were truly undertreated or were monitored without treatment within the accepted standards of care. This uncertainty regarding the composition of the types of DME patients in this study, however, does not influence the study findings that when patients were deemed to need treatment, they received considerably less intensive therapy (either with focal laser or anti-VEGF injections) than their clinical trial counterparts.

A central reason for conducting this study was to broadly define how health care resource utilization had changed for all DME patients over the near decade of observation and specifically, assess how ancillary test use has changed. Previously a more broadly defined group of all macular edema patients were shown to have less than a 20% annual probability of having fundus photography or a fluorescein angiography performed from 2005 to 2009, both of which decreased from near 40% in 2001.[14] This study found that the rate of fluorescein angiographies per office visit showed no change over the observation periods assessed. Additionally the percentage of patients who had an FA increased in each of the successive 2-year cohorts, as did the average number of FAs per patient who had at least one FA. The rate of patients with fundus photography was unchanged over the study as well. Together this data suggests that the previously reported declining trend in ancillary test usage may not pertain to the more selective group of DME patients.

Additional limitations of this study need to be noted. First, again due to not having chart level data, diagnoses were unable to be verified. To lessen this issue, very strict exclusion criteria was applied such that all patients who have a diagnosis that maybe confused with DME or could influence rates of resource usage (ie. OCTs for glaucoma) either before or after the index date were removed. Previous reports either did not include as extensive a list of exclusion diseases or only removed previous diagnoses, not diagnoses during the observation period. Also these reports allowed for the inclusion of proliferative diabetic retinopathy patients who may receive anti-VEGF therapy for reasons other than DME. This difference highlights a benefit of using a large claims database, in which considerable numbers can be maintained for study regardless of the strict inclusion/exclusion criteria.

An additional limitation is that the data in this study is limited to the end of 2012, and may not reflect changes that have occurred in more recent years. One example is this is the lack of aflibercept use in this dataset. With the recent release of Protocol T data, this DME treatment will continue to evolve of which aflibercept is likely to play a much more prominent role.[17] It is unclear how trends in care would be altered if the rates of ranibizumab or aflibercept were different. In addition, since the data for this study comes from a single insurer and may not reflect trends for DME patients covered by other insurances or other entities (the Veterans Affairs Health System for example). Nor are we able to rule out the possibility that anti-VEGF usage was paid for by patients in an out-of-pocket manner, but given the high costs of the medications, we feel this is very unlikely to represent a significant number of treatments. Lastly, counts were complied on a per/patient basis and not a per/eye basis. Clearly any change in the proportion of the bilateral patients over the observational cohorts could influence the relative rates of resource usage with the study. Of note however, this also further underscores the possibility of patients being treated less frequently then their clinical trial counterparts as anyone with two eyes being treated should have increased the number of treatments, yet the average rate of injections remained low.

The aim of this study was to obtain a more complete picture of how the care and resources used for the management of diabetic macular edema has changed in a national sample. The findings of this study suggest a significant number of DME patients may be under treated, raising concern that visual potential in these patients is not being maximized. Future work will be needed to determine how these trends continue to evolve and if greater acceptance of anti-VEGF therapy for DME occurs.
